# Attitudes and cognitive distances: On the non-unitary and flexible nature of cognitive maps

**DOI:** 10.2478/v10053-008-0140-y

**Published:** 2013-09-20

**Authors:** Claus-Christian Carbon, Vera M. Hesslinger

**Affiliations:** 1 Department of General Psychology and Methodology, University of Bamberg, Germany; 2 Bamberg Graduate School of Affective and Cognitive Sciences, Germany

**Keywords:** cognitive geography, cognitive distortions, cognitive map, heuristics, social attitudes, continental drift, Obama, mental wall, distance estimations, distortion, bias

## Abstract

Spatial relations of our environment are represented in cognitive maps. These cognitive maps are prone to various distortions (e.g., alignment and hierarchical effects) caused by basic cognitive factors (such as perceptual and conceptual reorganization) but also by affectively loaded and attitudinal influences. Here we show that even differences in attitude towards a single person representing a foreign country (here Barack Obama and the USA) can be related to drastic differences in the cognitive representation of distances concerning that country. Europeans who had a positive attitude towards Obama’s first presidential program estimated distances between US and European cities as being much smaller than did people who were skeptical or negative towards Obama’s ideas. On the basis of this result and existing literature, arguments on the non-unitary and flexible nature of cognitive maps are discussed.

## Introduction

Since introduced by Tolman (1948), the term *cognitive map* has been adopted by numerous disciplines such as psychology, behavioral sciences, computer science, and geography. This manifold usage has, however, led to some conceptual obscurity as the definitions referred to (if provided at all) were not always consistent (for details, see Hannes et al., 2012; Kitchin, 1994). In support of clarity, we will limit the following reflection to a lean conception that understands *cognitive maps* as cognitive representations of spatial (locational) information in terms of land-marks, their relative positions, and distances between them. This is in line with the original definition given by Tolman (who talks of a “cognitive-like map of the environment … indicating routes and paths and environmental relationships”, p. 192) and with the condensed meaning offered by Eysenck, Ellis, Hunt, and Johnson Laird (1994), for instance. Following the account of Downs and Stea (1973), we further include attributive information, more precisely descriptions, and “affectively charged” (p. 315) evaluations concerning the represented spatial information. Even so, this quite plain conception remains partly ambiguous, and how we figure the nature of the cognitively represented “spatial information” in detail depends on our understanding of the term *map* itself again.

Analyzing different scientific approaches to cognitive maps, Kitchin (1994) identified four categories: Approaches assuming or stating that a cognitive map (a) *is* a cartographic map (“explicit statement”), (b) is *like* a cartographic map (“analogy”), (c) is used *as if it were* a cartographic map (“metaphor”), and (d) has *no literal meaning* (“hypothetical construct”). In our view, however, any usage of the term *map* will always be contaminated by the way it is typically used in everyday life: in the sense of a cartographic map. Thus, even if we follow the idea of a cognitive map as a metaphor or a hypothetical construct, we might involuntarily attach to misleading implications associated with the household word. Kuipers (1982) already noted that “…metaphors and images must be treated very carefully in scientific investigations, lest their accidental properties be confused with the real properties of the phenomenon being studied” (p. 203). We are here reminded of similar misconceptions arising, for example, from the computer-metaphor in cognitive psychology that, in the end, falsely implies a kind of serial processing of discrete information units. Concerning cognitive maps, potential misconceptions arising from a reference to the everyday usage of the term map include assumptions on unity and stability: An actual cartographic map has a consistent design, is metric throughout, and once it has been printed, no further changes can be made.

### Constructed from pieces: The non-unitary nature of cognitive maps

How plausible would it be at all to assume that cognitive maps are of a unitary nature? In order to answer this question, one might begin by going back to how our knowledge about environments is presumably acquired and developed. A quite popular notion in this regard is that of sequential progression as put forward by Siegel and White (1975), who propose that newly developed spatial representations advance from mere landmark knowledge in the beginning, to route knowledge, and finally to survey knowledge. Assuming the unity of the resulting cognitive representation or map would, at least, be plausible in the context of this framework. Ishikawa and Montello (2006), however, note that the framework itself has found empirical objection rather than support; and their own data, for instance, demonstrate that some persons are able to develop survey knowledge from the very beginning (i.e., after only one session of exposure to test environments).

Taking a constructionist perspective, Tversky (1993) argues that our knowledge about environments is potentially acquired and recalled piecemeal. Accordingly, the cognitive representation of complex, less well known environments in particular should not be or resemble “one single, coherent maplike cognitive structure” (p. 15) but should consist of “snippets of information” (p. 21) that stem from various sources and can have different forms (e.g., memory of direct experience, cf. “direct sources”, Montello, 1997; learned facts, etc.). Tversky therefore proposed to speak of *cognitive collages* instead of *cognitive maps* in this respect, thereby emphasizing that these representations are not (necessarily) coherent. Referring to various kinds of behavioral data gathered from previous research, Montello (1992) similarly argued that knowledge of the environment is not to be described by a uniform metric as it “is incomplete, distorted, asymmetric, discontinuous, and imperfectly coordinated” (p. 143). And Kuipers (1982) pointed to findings indicating that spatial knowledge can be represented in terms of disconnected cognitive components instead of one single map.

Theoretical considerations as well as empirical evidence favoring the notion that cognitive maps are arranged in a (partially) hierarchical fashion (Hirtle & Jonides, 1985; McNamara, 1986; A. Stevens & Coupe, 1978) obviously add further support to this line of argument. Hierarchical organization is just one of a number of distortive factors (like, inter alia, rotation and orthogonal alignment) that cause systematic errors in the cognitive representation of a given environment. The effect of a specific distortive factor does not necessarily concern this representation as a whole but can be limited to certain parts of it; moreover, the effects of different distortive factors are not consistent per se. In one way or the other, distortion will reduce or disrupt unity, coherence, or homogeneity (Tversky, 1993).

### Prone to change: The flexible nature of cognitive maps

Cognitive maps do not suddenly “pop up” in our cognitive apparatus: They are acquired through development (Downs & Stea, 1973), meaning through a time-demanding process (“spatial microgenesis”, Montello, 1998, p. 143). Even a relatively mature, refined map is prone to change. Transferring Boulding’s (1961) ideas on subjective knowledge (which he calls “image”) to cognitive maps, Downs and Stea stated, for instance, that incoming information can affect an established cognitive map in three different ways: It can (a) confirm it, (b) be added to it, or (c) induce reorganization.

From an evolutionary point of view it is indeed reasonable to assume that cognitive maps are inherently flexible, as stable cognitive maps would not be at all adaptive in an ever-changing world. Being inclined to “tune” and “update” a cognitive map with new information, in contrast, contributes to maintaining effective orientation and navigation even when contextual spatial conditions have changed — a crucial factor for surviving. So, the flexibility of the cognitive map means adaptivity (cf. Kaplan, 1987).

Flexibility is further implicated by interactionist approaches that expect reciprocal effects of (spatial) knowledge and behavior (Webber, Symanski, & Root, 1975) or (spatial) knowledge, behavior, and environment (Kitchin, 1996) to occur. From this point of view, insights about factors associated with the formation and quality of cognitive maps are especially interesting as they provide a basis for eventual interventions to improve spatial knowledge and related behavior (e.g., wayfinding). Findings that demonstrate positive effects of personal experience with specific environmental properties (e.g., Carbon, 2010b) and of active versus passive travelling and navigation (Chorus & Timmermans, 2010; Mondschein, Blumenberg, & Taylor, 2010) could thus be utilized to help individuals improve their wayfinding, navigation, or survey skills.

Some additional (indirect) empirical evidence for the flexibility of cognitive maps furthermore could be given by studies investigating the impact of personal involvement and attitudes on distance estimates. The key lies in the potentially flexible nature of personal involvement and attitudes themselves. Attitudes, for instance, can change or be changed due to repeated exposure (Zajonc, 1968) and active elaboration (Carbon & Leder, 2005a) as well as persuasion or emotional appeal (for an overview, see Olson & Zanna, 1993). Presuming that the resulting change will concern an attitude already shown to have an impact on the specific manifestation of a cognitive map, this map will most probably also be modified.

### Reprise: Attitudes and cognitive maps

Ekman and Bratfisch (1965) were the first to present data capturing the relationship of emotional involvement and subjective distances (see also Bratfisch, 1969; Stanley, 1971; Strzalecki, 1978). As subsequent research has confirmed, attitudes can be related to selective distortions (i.e., distortions that pertain to only some parts of a cognitive map, while others remain unaffected) as well. For instance, people with negative attitudes towards foreign states or continents showed overestimated trans-national/regional versus intra-national/regional (“mental wall”, Carbon & Leder, 2005b, p. 750) or trans-continental versus intra-continental distance estimations (“cognitive continental drift”, Carbon, 2010a, p. 715; “psychological plate tectonics”, Friedman & Brown, 2000, p. 218).

Importantly, attitudes do not have to directly concern a certain territory in order to find reflection in the associated cognitive map; attitudes concerning political aspects relating to a territory have been shown to do so as well. Carbon (2010a), for instance, asked European participants to estimate distances between cities in Europe and the USA. As the results revealed, participants who disliked the Iraq war started by the USA in 2003 but were at the same time positive towards US citizens in general selectively overestimated distances between Europe and the USA. Table 1 offers an overview including this and further examples of research on attitudinal factors and cognitive distance.

**Table 1.** Exemplary Studies Investigating Cognitive Distance in Relation to, or Dependent on, Attitudinal Factors

Original publication	Attitudinal factor	Assessment of cognitive distance	Major finding
Ekman and Bratfisch (1965)	EI in what might happen in the target cities	Pairwise comparisons of “subjective distances” from Stockholm as epicenter; the relation of smaller to greater distance for each pair expressed as a percentage	EI inversely proportional to the square root of cognitive distance
Stanley (1968, 1971)	EI in what might happen in the target cities (directly referring to Ekman & Bratfisch'fs, 1965, procedure)	Direct estimations of distances from Armidale (Australia) as epicenter two different kinds of instructions: "subjective" vs. "geometric" (i.e., distances "as the crow flies")	Inverse relationship between EI and geometric distance
Strzalecki (1978)	Own personal interest and EI in the target cities	Estimation of distances from Opole (Poland) as epicenter in relation to a given standard distance (defined as distance between Nicosia/Cyprus and Opole)	EI inversely proportional to the square root of cognitive distance for geometric distances ≤ approximately 5,000 km; for larger geometric distances EI increased with cognitive distances
Kerkman, Stea, Norris, and Rice (2004)	Attitude toward ethnic diversity in friends, cross-national mobility, and travelling	Estimation of the physical locations of major cities in Canada, USA, and Mexico	Biased estimates for Mexican cities negatively correlated with diversity orientation
Carbon and Leder (2005a)	Attitude towards German reunification in 1990	Direct estimations of several distances (in km) within former West vs. East Germany ("within distances") as well as distances crossing the former border between them ("across distances")	Negative attitude towards German reunification lead to systematically overestimated across, but not within distances (“mental wall”)
Carbon (2010a)	Attitude towards Iraq war in 2003 and US politics, US citizens, and the USA in general	Direct estimations of several distances (in km) within Europe and within USA ("within distances") as well as trans-Atlantic distances ("across distances")	Negative attitude towards Iraq war in combination with general positive attitude towards US citizens lead to systematically overestimated across, but not within distances ("cognitive continental drift")

*Note*. EI = emotional involvement.

In the present study, we aimed at investigating whether even attitudes towards just one (admittedly important) person who represents a system or country are reflected in a cognitive map related to this country. Shortly after the inauguration of Barack Obama as the 44th President of the United States in 2009, opinions and attitudes concerning his political aims were clearly split as the new president “polarized” people (Nicholson, 2012; Schier, 2010). While some assumed his pre-election promises to be nothing but hot air, others were quite euphoric about and trusting in the change Obama had announced during his election campaign (Winter, 2011). In the middle of this politically polarized situation, we asked Europeans to estimate distances between several cities in Europe and the USA, as well as Baghdad, in order to test the resulting cognitive maps for systematically differing distortions. Especially for distances between Europe and the USA we expected estimations given by people with a positive attitude towards Barack Obama to be smaller than those given by people having a negative attitude towards him (cf. Carbon, 2010a). The conceptual implications and practical relevance of the results will be discussed.

## Empirical study

### Method

#### Participants

Ninety-two participants (77 female, 15 male) recruited on the campus of a German university (Bamberg) volunteered for partial course credit. The mean age was 21.4 years, with a range from 19 to 39 years. Thirty-nine persons (*M* = 21.6 years; 33 female, six male) reported having a negative and 52 persons (*M*21.3 years; 44 female, eight male) reported having a positive attitude towards Barack Obama and his political visions; one person gave no information regarding any attitude. The groups did not differ with regard to distributions of age and gender (for further details on the sample, see the Results section and Table 2).

**Table 2.** Averaged Ratings of the Post-Study Items Regarding the Attitude Towards Barack Obama and His Politics Split by the Attitude Group

Item	*M*_neg_	*M*_pos_	*t*(90)	*p*-value	Cohen’s *d*
1. Obama has the potential to make history as one of the greatest US presidents.	2,0 (1,0)	4,0 (1,4)	8,21	< .0001	1,73
2. Obama will help to solve the economic crisis.	3,1 (1,0)	4,6 (1,2)	6,49	< .0001	1,37
3. Obama will manage to make peace in Iraq.	3,6 (1,3)	5,3 (1,2)	6,46	< .0001	1,36
4. Obama will keep his word on his pre-election promises.	3,1 (1,0)	4,8 (0,7)	8,80	< .0001	1,86
5. Obama will change the relationship between USA and Europe to an extremely positive one.	2,6 (1,0)	3,7 (1,2)	5,00	< .0001	1,05
6. Obama will help the “third world” to solve its fundamental problems.	3,7 (1,4)	5,0 (1,1)	4,99	< .0001	1,05
7. Obama will strongly contribute to solving the climate problems.	3,6 (1,3)	5,4 (1,0)	7,37	< .0001	1,55
Overall: Averaged ratings (Items 1-7)	3,1 (0,7)	4,7 (0,6)	12,0	< .0001	2,53

*Note*. Standard deviations in parentheses. Neg = negative attitude, pos = positive attitude towards Barack Obama.

#### Stimuli

As cities of interest, we specified six cities in the United States (Chicago, Houston, Los Angeles, Miami, New York City, and Seattle), six cities in Central and Western Europe (Berlin, London, Madrid, Paris, Rome, and Zurich), as well as one city located in Iraq (Baghdad). US and European cities were selected on the basis of two criteria:

1. The cities had to be highly familiar, which was assured by ratings of 149 participants assessed by a pre-study not linked to the present one (for details, see Carbon, 2010a).

2. The configuration of the selected US and European cities should cover a large portion of the US and European territory, respectively.

We further included Baghdad for two reasons: (a) to assure a parallel design to Carbon (2010a) to be able to compare the resulting data patterns, and (b) because Baghdad, as the capital of the Republic of Iraq, where the US started a military operation in 2003, serves as a proxy for US foreign policy.

The combination of 6 (European) + 6 (US) +1 (Baghdad) cities yielded 13 × 12 = 156 unidirectional distances (i.e., Berlin New York City [NYC] and NYC Berlin as psychologically distinct distances) and 78 bidirectional distances (i.e., Berlin NYC as geometrically equal distances, i.e., the same distance), respectively. Among the 78 bidirectional geometric distances, 30 distances can be labeled as *within distances* (6 × 5 / 2 = 15 distances between two different cities located within Europe, and 15 distances between two different cities located within the USA) while 36 distances can be labeled as *across distances*, each between a European and a US city. The 12 remaining distances were labeled as *Baghdad distances* (six distances between a European city and Baghdad plus six distances between a US city and Baghdad).

#### Procedure

Participants were asked to estimate in kilometers all possible straight-line distances (as the crow flies) between the 13 cities contained in our selection; more precisely for both directions (e.g., Berlin-NYC and NYC-Berlin). In sum, each person estimated 156 unidirectional distances. After this test period (which lasted 20 min on average), the participants answered a series of seven questions about their attitude towards Barack Obama, the 44th President of the United States of America. The answer for each question (see Table 2) was captured by use of a 7-point Likert-scale (1 = *strongly disagree*, 7 = *strongly agree*). It is important to note that the complete data was collected within only one month (till 19th February 2009) after Obama’s presidential inauguration in 2009 (20th January 2009).

### Results and discussion

Before testing specific hypotheses, we checked whether there were significant differences between the unidirectional distances of the possible pairs of cities (e.g., Berlin NYC vs. NYC Berlin) by running a dependent measure *t*-test. As indicated by the result of the *t*-test, the direction given when asking for the distance between two cities did not have an effect on participants’ estimates, *t*(77) < 1.0, *p* = .4881, *ns*. This result is in line with the literature as the location of our participants (Bamberg) itself was not included in the set of cities (see studies on “reference points”; McNamara & Diwadkar, 1997). As a Pearson correlation analysis additionally revealed an extremely high interrelationship between the corresponding distance estimates in the two given directions (*r* = .996, *p* < .0001), we decided to collapse unidirectional distances to calculate all further analysis exclusively on the basis of bidirectional distances.

Depending on their individual attitude scores, we assigned participants to one of two “attitude groups”: For each participant we ave-raged the ratings s/he had given for the seven items reflecting different dimensions of their attitude towards the US president. Participants with a mean score smaller than 4 were classified as having a negative attitude towards Obama, whereas participants with a mean score of 4 and higher were classified as having a positive attitude towards him. As can be seen in Table 2, the positive attitude group differed significantly from the negative attitude group not only in the mean score (i.e., the split criterion) but in *each* of the seven items. This underlines the presence of multi-dimensional significant attitudinal differences between these two groups.

**Figure 1.**
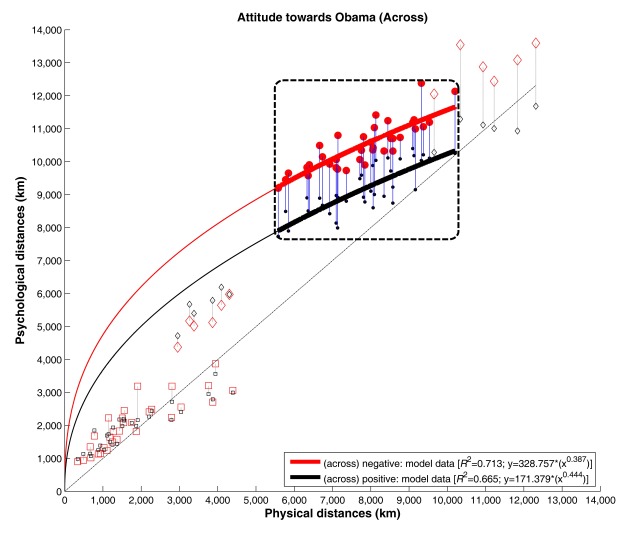
Bivariate scatterplot for psychological (cognitive) versus geometric (physical) distances split by distance category, and attitude towards Barack Obama (negative: red, positive: black data points). Curve fittings are calculated for across distances (between Europe and the USA) only. The distance data is organized according to the following distance categories: (a) *Baghdad distances* (indicated by diamonds) with Baghdad-Europe and Baghdad-USA (with distances < 6,000 km corresponding distances between Baghdad and Europe, while the other distances were between Baghdad and the USA), (b) *within distances* (indicated by squares) with two cities both located in Europe or both located in the USA, and (c) *across distances* (indicated by dots) which are distances between one city in Europe and another in the USA.

The distance data, corrected by excluding typical outliers (i.e., distances < 100 km as well as distances > 28,000 km; 5.60% of all distances were detected as outliers: 3.29% in the negative attitude group and 7.33% in the positive attitude group), were split by the five main distance categories: Baghdad-Europe, Baghdad-USA, Europe-Europe, Europe-USA, and USA-USA. As we were particularly interested in specific attitude-related distance distortions, we additionally split the data by attitude group (positive vs. negative attitude towards Barack Obama). As Figure 1 shows, distance estimations given by participants in the positive versus negative attitude group clearly differed from each other. The difference was especially pronounced in the “across” distances (distances between Europe and the USA), but it could also be found in distances between Baghdad and the USA.

The main hypothesis (that people who are positive towards Barack Obama and his political promises give shorter estimations for “across” distances than people with a negative attitude towards him) was tested via a two-step process. First, all five distance categories were tested for differences between the attitude groups via *t*-tests. Second, we con-ducted regression analyses for the “across” distances to get deeper insights into the relationship between the attitude towards Barack Obama and these distance estimations.

**Figure 2.**
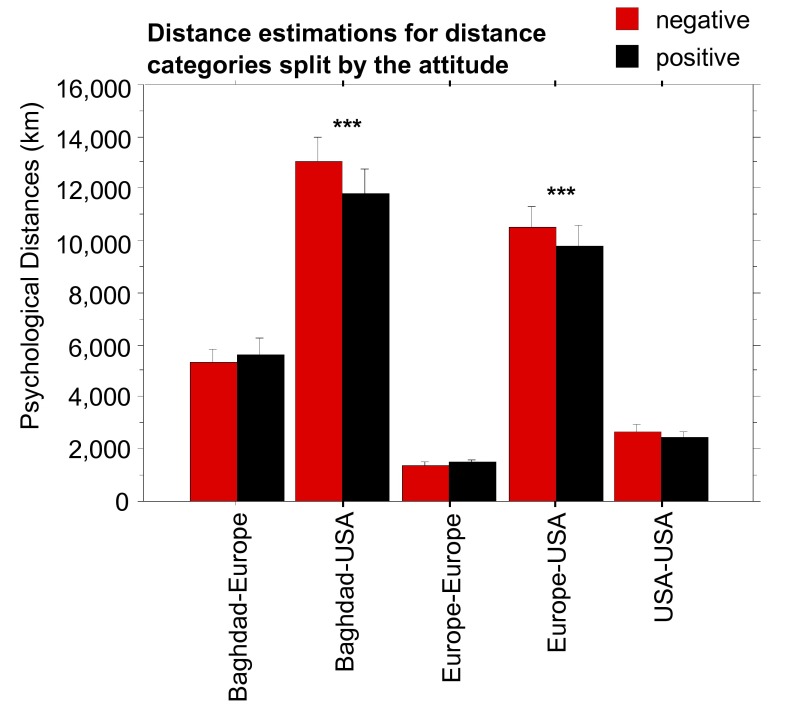
Distance estimations for the five distance categories split by attitude towards Barack Obama. Significant differences between the positive and negative attitude groups are indicated by asterisks (*** equals *p* < .0001). Error bars display ±1 standard error of the mean (*SEM*).

Regarding the mean distances for both attitude groups, we could indeed reveal significant differences between them for the distance categories Europe-USA, *t*(35) = 17.28, *p* < .0001, Cohen’s *d* = 2.92, and Baghdad-USA, *t*(5) = 15.51, *p* < .0001, Cohen’s *d* = 6.94. None of the other distance categories showed significant effects (see also Figure 2). Concerning distances between Europe and the USA as well as distances between Baghdad and the USA, the same relation with the attitude towards Obama was found: Participants with a positive attitude towards him estimated the trans-continental distances to be shorter than did people with a negative attitude towards him (*M*_diff_ = 1,338.5 km and 1,880.3 km,respectively).

**Figure 3.**
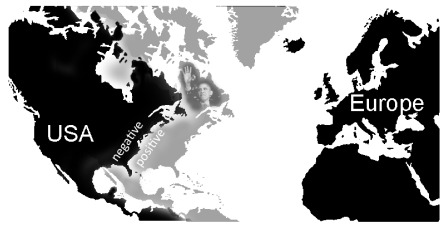
Illustration of the “Obama effect”: Pronounced differences (approximately 1,300 km) in estimations of across distances (between European and US cities) between persons with positive versus negative attitudes towards Barack Obama and his political visions.

To get deeper insights into the attitude-distance relation, especially for the across category “Europe-USA”, we submitted these data to regression analyses. As shown by the pioneering work of S. S. Stevens and Galanter (1957) and followers in the domain of cognitive distance research (e.g., Künnapas, 1960), the psychophysical function for large-scale distances fits very well with a power function of the type *y* = *a* × *x*^*b*^, with *y* being the psychological distance, *a* the scaling constant of the function, and *x* the geometric distance, while *b* provides the curvature of the function. In Figure 1, focused data on across distances are emphasized by solid data points encompassed by a dashed rectangular window. All the distance estimations of the positive attitude group are lower than those of the negative group (see Figure 3). Furthermore, the fit of the data of both attitude groups with power functions was very good, *R*_pos_ = .816 (*p* < .0001) and *R*_neg_ = .845 (*p* < .0001), respectively; the explained variances were very similar to comparable studies, (e.g., .828 *R*_across_ .843 in Carbon, 2010a). The exact curve functions can be retrieved from Figure 1. The fitted curves also show that the difference between the positive and negative attitude group was quite constant across the enquired distances that ranged from 5,582 km (London NYC) to 10,200 km (Los Angeles [LA] Rome). This descriptive result was further validated by setting both geometric distances as x in the curve equations, which resulted in a difference of 1,368 km (positive: 7,898 km; negative: 9,266 km) and 1,378 km (positive: 10,322 km; negative: 11,700 km), respectively, between the positive and negative attitude groups.

**Figure 4.**
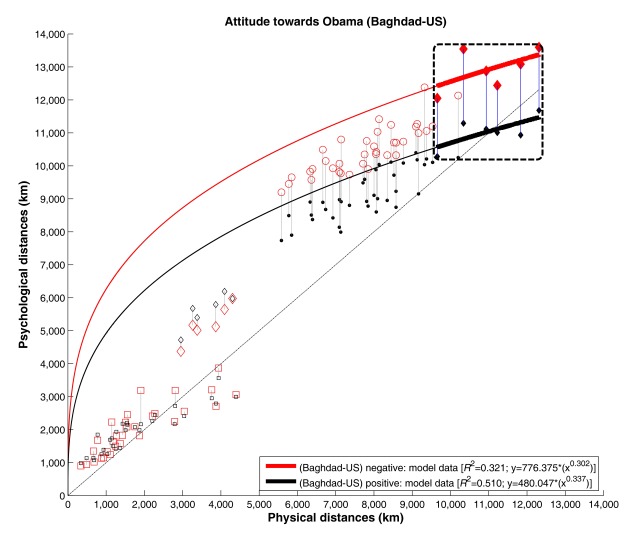
Bivariate scatterplot for psychological (cognitive) versus geometric (physical) distances split by distance category and attitude towards Barack Obama (negative: red, positive: black data points). Curve fittings are calculated for the distances between Baghdad and US cities only. As the plot is analogously generated to Figure 1, details can be read there.

With regard to distances between Baghdad and US cities, we observed a difference that was similar to the one already shown by the inference statistics above (cf. Figure 2). We therefore conducted parallel regression analyses on basis of a power function (see Figure 4). The difference between both attitude groups was again substantial, with the modeled difference being 1,829 km (positive: 10,572 km; negative: 12,401 km) for the shortest geometric distance (Baghdad NYC) and 1,872 km (positive: 11,475 km; negative: 13,347 km) for the longest geometric distance (Baghdad LA), respectively.

## General discussion

Inspired by previous research on the relationship of attitudes and cognitive distances, we utilized the historic event of Barack Obama’s polarizing inauguration in 2009 (Winter, 2011). Comparatively analyzing the cognitive maps of people with diverse attitudes towards the newly elected US president opened an ideal opportunity for testing whether the attitude towards one single person, namely the representative of a certain country, is reflected in the cognitive representation of spatial relations.

### Non-unitary and flexible: Conceptual considerations and implications for future studies

Participants were asked to estimate three different classes of distances: (a) distances between cities within Europe (Western Europe) and within North America (USA), respectively (*within distances*), (b) transcontinental distances between European and US cities (*across distances*),and (c) distances between Baghdad and European and US cities, respectively (*Baghdad distances*). In line with classical approaches (S. S. Stevens & Galanter, 1957) as well as with more recent literature having validated the psychophysics of large-scale distances, the obtained estimates of across distances of both attitude groups conformed to power functions. Importantly, compared to the negative attitude group, persons with a positive attitude towards Barack Obama uniformly showed lower estimations for all distances between European and US cities. Analogous distortion effects occurred for distances between Baghdad and US cities, while distances of the within category were not affected at all (see Figure 2). The scatter plots of the data (e.g., Figure 1) further indicate that distances for the different distance categories follow specific psychophysical functions. The respective pattern fits in very well with the hierarchical approach to the cognitive representation of space (Golledge, 1978; Hirtle & Jonides, 1985; McNamara, 1986) and points, like any kind of selective or regional distortion, to the (potential) incoherence or non-unitary nature of so-called cognitive maps. Considering this result, we share Tversky’s (1993) idea of cognitive representations of space resembling “collages” where different information layers, among them spatial relations, are gathered together without any strict overall coherence. The term *patchwork* used by Montello (1992) seems likewise appropriate.

The discovery that differences in attitude towards one single person are related to differences in cognitive large-scale distances also points to the high flexibility of cognitive maps: Of course, in the present case, the single person is the president of the Unites States, and as such, a person of ultimate importance for the political orientation of the whole nation. As the first representative of his country, Obama literally represents his country, and the attitude towards him might have operated in terms of a “halo effect” (Nisbett & Wilson, 1977). Another representative most probably will have another “halo”, so it is quite probable that a change on this level will be accompanied by changes in the respective cognitive map.

By using the present paradigm, however, we can only take a snapshot of this potential dynamic whole, and a range of questions is left open. In order to gain important further insights here, future studies should address the following points:

1. Most importantly, the causal direction of influences between attitudes and cognitive distances is to be investigated. We assume that attitudes towards one representative person do influence subjective distances involving the represented country. The opposite is just as likely: Subjective distances determine our attitudes. It is further possible (maybe even more likely) that the attitude-distance relation can be explained by one or more additional variables having equally directed effects on attitudes and subjective distances at the same time. In order to test these alternatives, experimental designs manipulating attitudes and subjective distances as well as potential third variables are needed.

2. We assume dynamic effects of attitudinal changes on our cognitive representations of the spatial environment. A proper investigation of this topic might be realized by means of a test-retest design manipulating attitudinal factors on a within-participant level.

3. In the present study, we dichotomized the attitude factor (positive vs. negative), so we could only test for a difference in cognitive distances among the groups. Whether the strength of attitude differences is further related to the magnitude of differences in distance estimations is still to be investigated.

### Coda: Distorted maps, biased behavior, and their benefits

Within a marketing framework, Swift (1999) showed that closeness between cultures directly affects liking, which can in turn lead to increased willingness to help when the respective other culture is in trouble (for different effects in personal space, see Nagel & Waldmann, 2013). Such effects have been observed with regard to natural catastrophes, for instance. The 2004 Indian Ocean earthquake that triggered a series of tsunamis killed approximately 230,000 people. Although Indonesia was hit by the most severe consequences (nearly 170,000 fatalities), most Europeans — being more familiar with Thai culture, cuisine, and tourism — particularly donated to Thailand more than to Indonesia. Similarly, major earthquakes in the years 2005 and 2008, each killing approximately 90,000 people, hardly attracted any interest, and the amount of donations was accordingly low as they affected areas situated in Pakistan and China, two regions Europeans are not so familiar with.

The present study showed that parallel to their attitudes towards Barack Obama, people differ in their estimations of distances between European and US cities. For people who believed that Obama would bring about the change he promised and effectively solve some fundamental problems (see Table 2) the subjective distances between the continents were smaller than for those who did not. Whether these smaller distances also imply a higher relevance of the US towards everyday European issues is a matter of conjecture.

Combining the aforementioned findings and considerations, an interesting perspective can be developed. The crucial points to bring together are the following:

1. Reduced subjective distance enhances helping behavior.

2. A positive attitude towards a single representative of a place can relate to the subjective distance to the respective place.

Facing a humanitarian disaster in an area that potential donators do not feel strongly related to, a much-loved, prominent figure acting as a representative of the suffering area might lend precious assistance. She or he might make people feel closer to those suffering, which will enhance the sense of responsibility felt as well as humanitarian behavior — an undeniably beneficial bias.
